# Acid Dye Removal from Aqueous Solution by Using Neodymium(III) Oxide Nanoadsorbents

**DOI:** 10.3390/nano10030556

**Published:** 2020-03-19

**Authors:** Shahin Ahmadi, Leili Mohammadi, Abbas Rahdar, Somayeh Rahdar, Ramin Dehghani, Chinenye Adaobi Igwegbe, George Z. Kyzas

**Affiliations:** 1 Department of Environmental Health, Zabol University of Medical Sciences, Zabol 986161588, Iran; sh.ahmadi398@gmail.com (S.A.); rahdar89@gmail.com (S.R.); 2 PhD of Environmental Health, Infectious Diseases and Tropical Medicine Research Center, Resistant Tuberculosis Institute, Zahedan University of Medical Sciences, Zahedan 9816743463, Iran; lailimohamadi@gmail.com; 3 Department of Physics, Faculty of science, University of Zabol, Zabol 538-98615, Iran; 4 Department of Environmental Health, Kerman University of Medical Sciences, Kerman 7616913555, Iran; m.heidarimokarrar@gmail.com; 5 Department of Chemical Engineering, Nnamdi Azikiwe University, Awka 420218, Nigeria; ca.igwegbe@unizik.edu.ng; 6 Department of Chemistry, International Hellenic University, 654040 Kavala, Greece

**Keywords:** acid blue 92, response surface methodology, adsorption, neodymium(iii) oxide, central composite design, water treatment

## Abstract

In the current work, neodymium oxide (Nd_2_O_3_) nanoparticles were synthesized and characterized by means of X-ray diffraction (XRD), Fourier-transform infrared spectroscopy (FTIR), and scanning electron microscopy (SEM). The major aim/investigation of this research was to fit/model and optimize the removal of Acid Blue 92 (AB92) dye from synthetic effluents (aqueous solutions) using the adsorption process based on neodymium oxide (Nd_2_O_3_) nanoparticles. To optimize the adsorption conditions, central composite design (CCD) based on response surface methodology (RSM) was applied. The effects of pH (3–9), adsorbent dosage (0.1–1 g/L), initial concentration of AB92 (100–300 mg/L), and contact time (10–100 min) on the adsorption process were investigated. Apart from equilibrium and kinetic experiments, thermodynamic evaluation of the adsorption process was also undertaken. The adsorption process was found to have the best fitting to Langmuir isotherm model and pseudo-second-order kinetic equation. Also, the process was found to be spontaneous and favorable with increased temperature. The optimal conditions found were: pH = 3.15, AB92 concentration equal to 138.5 mg/L, dosage of nanoadsorbent equal to 0.83 g/L, and 50 min as contact time, which resulted in 90.70% AB92 removal. High values for the coefficient of determination, *R*^2^ (0.9596) and adjusted *R*^2^ (0.9220) indicated that the removal of AB92 dye using adsorption can be explained and modeled by RSM. The Fisher’s *F*-value (25.4683) denotes that the developed model was significant for AB92 adsorption at a 95% confidence level.

## 1. Introduction

It is generally accepted that dyestuffs are very hazardous species of industrial effluents and need to be treated [1,2,3]. Azo dyes possess 70% of the total dyes in the world. Due to the presence of azo bonds (–N=N–), sulfonic groups and aromatic rings in dye compounds, azo dyes are hardly decomposed in the natural environment and have toxic, mutagenic, and carcinogenic effects [4]. They are also unwanted compounds in the environment because they reduce the penetration of light and impair the process of photosynthesis [5,6]. In addition, these compounds can cause negative effects on the appearance and quality of water [6]. Therefore, wastewaters containing this type of dyes were highlighted as one of the most important threatening factors in environmental and public health [2,5]. Different studies have reported that there are several methods for removing dyes from textile wastewaters, including the application of photodecomposition [6], electrolysis [7,8], adsorption [9,10,11,12,13,14,15,16,17,18], oxidation [19], biodegradation [20], combined sonochemical and adsorption [21], coagulation-flocculation [22,23], etc.

Adsorption is widely used because of its relatively simple design, low cost, and removal of color and other pollutants with great efficiency [19,24,25,26,27]. Adsorption can be either physisorption (which involves fairly weak (not strong enough) intermolecular forces), or chemical sorption (namely chemisorption), which involves the creation of chemical bonds among the funtioanl groups of pollutants and the surface of the adsorbent materials [20]. Activated carbons have been used successfully to remove organic and mineral pollutants [28]. Recently, nanotechnology has become known as a key and effective technology in science, technology, and industry [29]. Nanoparticles have been found to have high potential in adsorption of organic compounds. Last years, it is great of interest the removal of colors/dyes from wastewaters or/and sewage tanks by adsorption onto nanoparticles because they have a higher surface/volume ratio than other adsorbents [30,31,32].

Recently, more attention has been given to the applications of the adsorbents prepared by several rare-earth metals (REMs) for water treatment due to their favorable chemical properties. Compared to typically used metals (e.g., iron, aluminum, and manganese), the adsorbents prepared by the REMs have more functional groups on their surfaces [33], and better catalysis reaction performance [34], which are favorable for arsenic uptake. In addition, they have no or lower toxicity for humans. A series of REM-based adsorptive materials in the form of metal oxides/hydroxides, metal oxide/hydroxide modified adsorbents and metal ion impregnated adsorbents have been recently reported for the effective decontamination of arsenic. One of these rare earth elements is neodymium (Nd), with atomic number 60, atomic radius 1.821 A, valence state +3 and electronic configuration 4f^4^6s^2^. Also, it has a noteworthy abundance in the upper Earth’s crust (26 ppm), which is 3^rd^ in order after Ce (64 ppm) and La (30 ppm) [35].

Neodymium(III) oxide (Nd_2_O_3_) nanoparticles are widely used as coloring agents for ceramics and glasses, catalysts, raw materials of the neodymium alloy and neodymium metal, and also as dopants for high-efficiency solid-state lasers due to their unique thermal and physicochemical properties [36,37]. However, there is no special mention of the possible use of Nd_2_O_3_ nanoparticles as materials for dye adsorption. There is only mention of the adsorption of salicyl hydroxamic acid on Nd_2_O_3_ surfaces [38], as well as in another study NdCl_3_ was incorporated into order mesoporous carbon (OMC) through an incipient wetness technique to enhance sunset yellow removal [39]; however the latter is a modification of OMC and not a “clear” use of Nd_2_O_3_ as adsorbent.

The major target of the present study is to model the reduction of AB92 dye from aqueous solutions by adsorption onto Nd_2_O_3_ nanoparticles synthesized and also optimized in experimental conditions. The central composite design (CCD) via the response surface method (RSM) was employed to design the experimental runs for AB92 adsorption. It is an important tool applied for the development and optimization of novel processes, and improvement of existing processes [40,41,42]. The RSM was used to study the influence of the operating parameters (solution pH, Nd_2_O_3_ nanoparticles dosage, AB92 concentration, and time of reaction) and their relationships or interactions in order to maximize the efficiencies of adsorption and to determine the optimal conditions. The isotherm, kinetics, and thermodynamics of the process were also explored.

## 2. Materials and Methods

### 2.1. Materials

**Figure 1:**
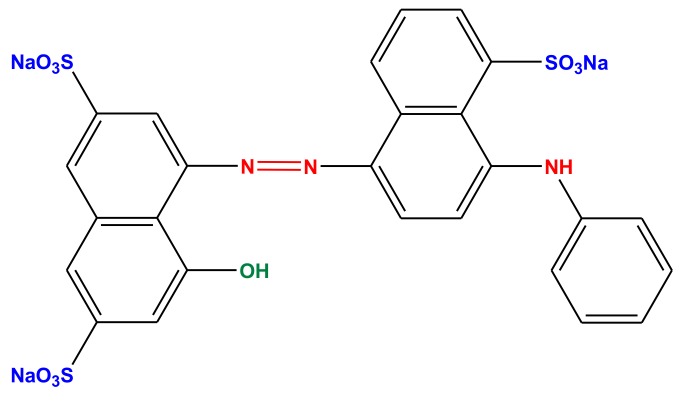
Structure of Acid Blue 92 (AB92).

AB92 (Acid Blue 92) dye was purchased from AlvanSabet Corporation (Hamadan, Iran) and used as model pollutant. AB92 (anazolene sodium, C_26_H_16_N_3_Na_3_O_10_S_3_) is a commercial salt consisting of a mixture of dye and an inert product; the true dye content was 40%. This dye purity was taken into account in calculating the concentrations. Figure 1 shows the chemical structure of AB92. The molecular weight of the dye was 695.58 g/mol. AB92 is a monoazo compound bearing three sulfonic groups; it holds negative charges in aqueous solutions. Dye solutions were prepared by means of direct dilution in water (at the appropriate concentration) and the pH was controlled with hydrochloric acid and sodium hydroxide solutions.

All other reagents (sodium hydroxide (NaOH, 98%), and sulfuric acid (H_2_SO_4_, 99.99%)) were of analytical grade and purchased from Merck (Berlin, Germany). All solutions were prepared using deionized water. The pH of the solution was adjusted by micro-additions of HCl (0.1 N) or NaOH (0.1 N) solutions.

### 2.2. Synthesis of Nd_2_O_3_ Nanoadsorbent

The synthesis of Nd_2_O_3_ nanoparticles were carried out based on a published study [43]. The ligand Schiff base was used to synthesize the nanoparticles of neodymium. At first, 0.06 mol of 2-hydroxy-1-netaldehyde were dissolved in 30 mL of methanol using magnetic stirrer (0.5 h), next 0.03 mol of 1,4-diaminobutane were diluted in 30 mL of methanol in another container similarly by using magnetic stirrer (0.5 h) MSL 50 Digital (VELP Scientifica Srl, Usmate, Italy) and then added to the previous solution drop-by-drop. Subsequently, an appropriate amount of Nd(NO_3_)_3_∙6H_2_O (with a molar ratio of 1:4) was dissolved in the above solution (magnetic stirring 2 h). The solution was then stored at a reflux condition for 3 h. The compound formed was washed with distilled water and methanol (Soxhlet apparatus, 12 h) and dried at 60 ℃ for 3 h (oven). The ligand Schiff base was used as the making base. It was combined with salt with a different molar ratio (in a mortar). Then, it was calcified for 5 h in a furnace at 900 ℃.

### 2.3. Characterizations

Fourier-transform infrared spectroscopy (FTIR) spectra were taken on a JASCO 640 plus machine (4000–400 cm^−1^) (Zeiss, Berlin, Germany) at room temperature so as to reveal the functional groups of Nd_2_O_3_ nanoadsorbents, which contributed to the removal of AB92. The morphology of nanoparticles was evaluated by taking scanning electron microscope (SEM) images using the LEO instrument model 1455VP (Zeis, Jena, Germany). XRD patterns were obtained with a diffractometer of Philips Company (PANalytical B.V., Almero, The Netherlands) with X’Pert Pro monochromatized CuK*α* radiation.

### 2.4. Adsorption/Desorption Experiments

The adsorption experiments were based on multi-parametric study. The effect of pH was studied in the range of 3–9, as well the influence that has the increase of nanoadsorbent’s dosage from 0.1 to 1 g per 1 L of effluents. Also, the kinetic behavior of the process was examined running experiments varying the contact time between sorbent and sorbate from 10 to 100 min, and the same was done for the determination of initial concentration of AB92 (100–300 mg/L). The preparation of AB92 stock solution was carried out by using double-distilled water. Batch experiments were performed in 250-mL Erlenmeyer flasks. The desired initial concentrations of dye were added to the flasks with pipettes, while the pH of the solution was adjusted by micro-additions of 0.1 N HCl or 0.1 N NaOH. The initial solution pH was analyzed with a MT65 pH-meter (Mettler-Toledo GmbH, Giessen, Germany). Then, a fixed nanoadsorbent’s mass (Nd_2_O_3_) was added to the flask (already containing the respective dye solution of final volume of 100 mL). The flasks were placed to the thermostatted bath and agitated (*N* = 180 rpm) for 2 h.

The initial and residual (final) dye concentrations in solutions were measured by using an ultraviolet (UV)-visible spectrophotometer (Shimadzu, Model CE-1021, Columbia, MD, USA) at *λ*_max_ = 620 nm (wavelength of maximum absorbance). The percentage of adsorption was calculated as follows (Equation (1)):

(1) $$Removal(\%)=\left(\frac{C_{0}-C_{f}}{C_{0}}\right)\cdot 100\%$$

where *C*_0_ (mg/L) is the initial AB92 dye concentration and *C_f_* (mg/L) is the final concentration.

Desorption experiments were performed in batch mode using constant (optimal) adsorption conditions found from experimental design (pH = 3.15, AB92 concentration equal to 138.5 mg/L, dosage 0.83 g/L, 50 min as contact time, 25 °C, *N* = 180 rpm) and divided into two main categories: (i) desorption experiments for finding the optimal eluent, and (ii) reuse cycles with continuous adsorption-desorption experiments. At first, after the end of adsorption stage, the adsorbent materials were separated from supernatant using filtration membranes. Then, the adsorbent particles separated were placed in flasks using deionized water as eluent with pH-adjusted values (10, which is the reverse pH conditions as those of adsorption to break the adsorption forces and then desorb). The desorption step (as in adsorption step) lasted 24 h. The quantitative evaluation of desorption was done using desorption percentages, calculated from the difference between the loaded amount of drugs on adsorbent after adsorption and the amount of drugs in solution after desorption. To investigate the reuse ability of adsorbents, the above procedure with the same conditions (firstly adsorption and then desorption) was repeated 5 times.

### 2.5. Experimental Design and Statistical Analysis

Central composite design (CCD) was done using Design Expert software 7.1 (Stat-Ease, Minneapolis, MN, USA). 4-factors at 3-levels (3^4^) of full factorial were selected for this study. The CCD in RSM was used to generate the experiments with 30 experimental runs which comprise of 8 axial points, 16 factorial points, and 6 replicates at the center points. The optimization studies were carried out by studying the effects of pH, initial AB92 concentration, Nd_2_O_3_ dosage and time (independent variables). The chosen independent variables for this study were coded according to Equation (2) [44]:

(2) $$x_{i}=\left(\frac{x_{i}-x_{0}}{\Delta x}\right)\cdot 100\%$$

where *x_i_* is the dimensionless coded value of the independent variable, *x*_0_ is the value of *x_i_* at the center point and Δ*x* is the step change value. The behavior of the system is explained by the following empirical second-order polynomial model (Equation (3)):

(3) $$Y=\beta_{0}+\sum_{i=1}^{k}\beta_{i}x_{i}+\sum_{i=1}^{k}\beta_{ii}x_{i}{}^{2}+\sum_{i=j}^{k-1}\sum_{i=j+1}^{k}\beta_{ij}x_{i}x_{j}$$

where *Y* is the predicted response, *x_i_*, *x_j_*, …, *x_k_* are the input variables, which affect the response *Y*, *x*_2*i*_, *x*_2*j*_,…, *x*_2*k*_ are the square effects, β_0_ is the intercept term, *x_i_x_j_, x_j_x_k_* and *x_i_x_k_* are the interaction effects, β_i_ (i = 1, 2, …, *k*) is the linear effect, β_ii_ (i = 1, 2, …, *k*) is the squared effect, *β_ij_* (*j* = 1, 2, …, *k*) is the interaction effect and Σ is the random error [45].

The experimental range and levels of the independent variables used in the present study are stated in Table 1. The experimental data obtained were fitted to the empirical second-order polynomial regression model (Equation (3)). Also, the coefficient of determination (*R*^2^) value was compared to the adjusted *R*^2^ and predicted *R*^2^ values to check the adequacy of the model. The analysis of variance (ANOVA) was used to examine the interactive effects of the process variables on the AB92 adsorption efficiency. The *R*^2^ is used to determine the potential of a regression model to predict a process. The adjusted *R*^2^ is applied to assess the goodness-of-fit of the model while the predicted *R*^2^ is used to define how good a model predicts a process.

## 3. Results and Discussion

### 3.1. Techniques of Characterization

**Figure 2:**
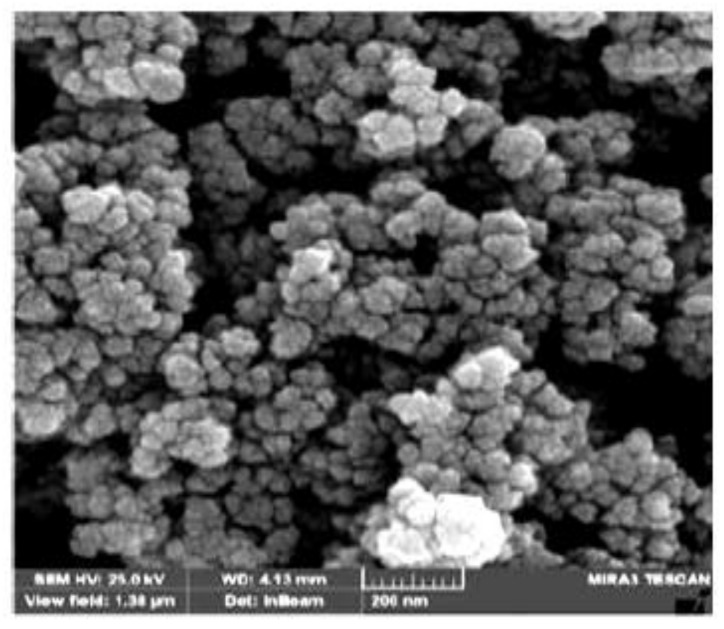
Field-emission scanning electron microscope (FE-SEM) image of the Nd_2_O_3_ nanoparticles.

The structural surface of the Nd_2_O_3_ nanoparticles was visualized using the SEM technique. Figure 2 shows the SEM image (150 k*x*) of the adsorbent which seems to be approximately spherical-like in structure forming nanoclusters. The Nd_2_O_3_ sample consists of irregular shapes with pores of varying sizes, which will make available the active sites for the adsorption process and the take in of the AB92 dye particles. Moreover, it is fact that SEM images shows some percentage of agglomeration, but this does not imply that there is any porosity. Due to agglomeration, some surface area will be reduced so the adsorption capability will probably be affected. However, adsorption is a combined and in the case of oxides is majorly explained with attractive bonding (forces) and not so much with simple deposition on the surface area.

**Figure 3:**
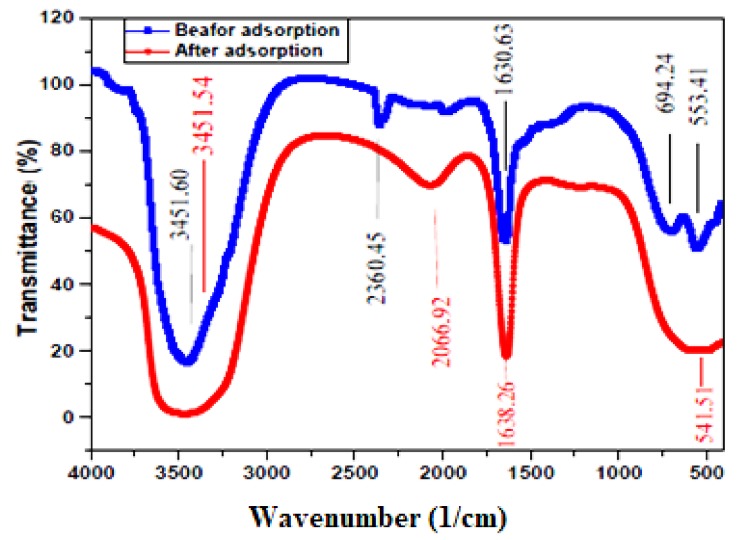
Fourier-transform infrared spectroscopy (FTIR) spectra of the Nd_2_O_3_ nanoparticles before adsorption (up) and after adsorption (down).

FTIR spectroscopy showed the functional groups present in the nanoadsorbent (Nd_2_O_3_ nanoparticles). The FTIR spectra of the nanoparticles (before and after dye adsorption) were recorded in the range of 400–4000 cm^−1^ (Figure 3).

**Figure 4:**
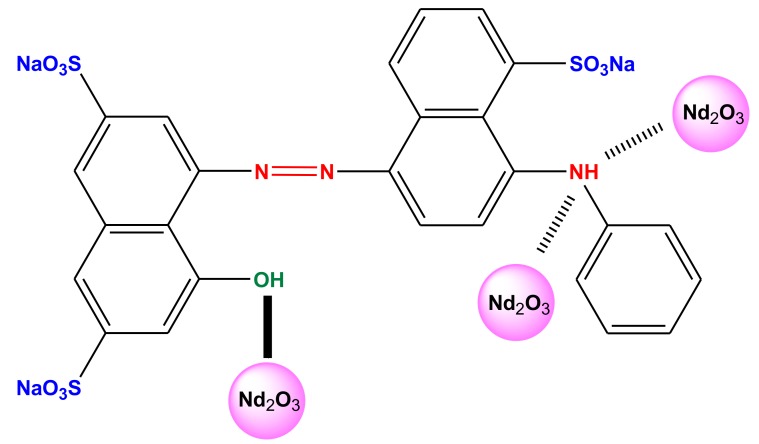
Proposed adsorption mechanism of AB92 and Nd_2_O_3_ nanoparticles.

In the case of Nd_2_O_3_ nanoparticles before dye adsorption, the presence of alkyl halides (C–Br stretching) was confirmed, as well as that of alkynes (–C≡C–H with C–H bend) and primary amines (N–H) (originating from the dye molecule—Figure 1) bending which were revealed at the band regions of 553.41, 694.24 and 1630.63 cm^−1^, respectively. An absorption peak at 2360.45 cm^−1^ was also observed which corresponds to –C=C– stretching of alkynes (a very weak band). The peak of 3451.54 cm^−1^ (very broad and strong band) is credited to O–H stretch, H–bonded (alcohols, phenols). This strong band (O–H stretch) participated actively in the adsorptive removal of AB92. After AB92 adsorption, the intensities of the bands were reduced from 553.41 and 1630.63 cm^−1^ to 541.51 and 1638.26 cm^−1^, respectively. The band intensity of 2360.45 was decreased to 2066.92 cm^−1^. Also, the intensity of the O–H band was increased from 3451.54 to 3451.60 cm^−1^. This peak shift implied the interaction of the adsorbate with the functional groups of the adsorbent [46]. Based on the above FTIR spectrum a proposed adsorption mechanism can be illiustrated in Figure 4.

**Figure 5:**
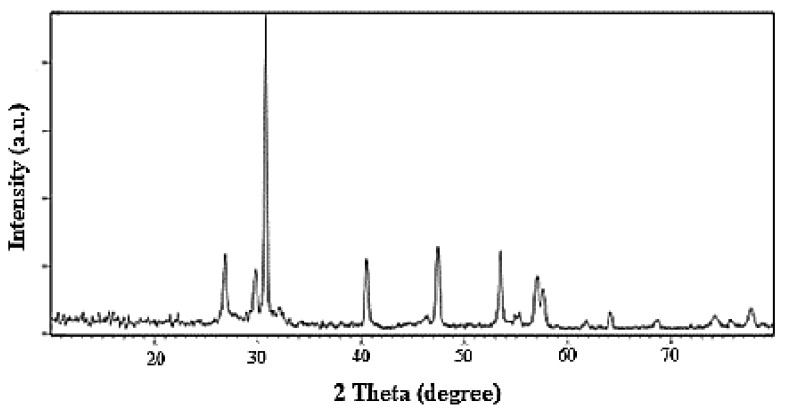
X-ray diffraction (XRD) pattern of the Nd_2_O_3_ nanoparticles.

The X-ray diffraction (XRD) pattern of Nd_2_O_3_ nanoparticles is depicted in Figure 5.

The XRD pattern indicates that the maximum peak is around 2*θ* = 31° with high intensity. This XRD pattern indicates that a crystalline nanoparticle has been prepared and no remarkable impurities were seen in the material, implying high purity of the nanoparticle. From the XRD data, the average crystallite size (*D_c_*) of the nanocomposite was calculated using the Scherer equation:

(4) $$D_{c}=\frac{K\cdot \lambda }{\beta \cdot \cos\theta }$$

where *λ* (nm) is the wavelength; *K* (usually 0.9) is the so-called shape factor and *β* is the breadth of the observed diffraction line at its half maximum intensity. The average size, *D_c_* of the Nd_2_O_3_ nanoparticles was evaluated to be 83 nm.

### 3.2. Model Fitting—Statistical Analysis—Adsorption Optimization

**Figure 6:**
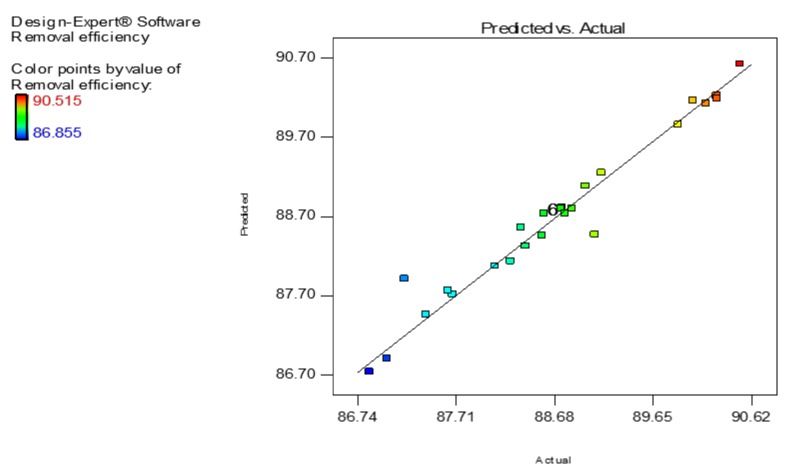
Predicted values versus experimental (actual) values of AB92 adsorption.

The experiments were carried out using the experimental conditionn described in Table 2. The experimental data generated were analyzed by using special software (Design expert software, Stat-Ease 7.1 trial version, Minneapolis, MN, USA). The results were analyzed via the RSM to acquire the empirical model. The actual and predicted adsorption percentages are shown in Table 2. The experimental values were established to be close to the predicted responses obtained for a particular run (Figure 6 and Table 2). The experimental data were examined via the sequential model sum of squares and model summary statistics to find the most appropriate models. Table 3 shows that the quadratic model gives the highest *R*^2^, adjusted *R*^2^ and predicted *R*^2^ values compared to other models (linear, interactive, and cubic) apart from the cubic model. The latter happens because a cubic model cannot be applied to fit/model the data since it is aliased. An aliased model means that it is a result of insufficient experimental runs to independently appraise all the models [47].

Table 4 shows the ANOVA results. ANOVA is a statistical method that divides the total variation in a set of data into component parts linked with particular sources of variation for the intention of testing the hypothesis on the parameters of the model [48]. The ANOVA shows that the second-order polynomial model (quadratic model) developed was statistically suitable for the analysis, representation, and explanation of the AB92 adsorption process at the variables’ studied range. The *F*-value was used to check the significance of the regression coefficients. The *p*-values were used to check the significance of each of the interactions among the variables, which may indicate the patterns of the interactions among the variables. A *p*-value less than 0.05 shows the influence and significance of a term [49].

After analysis, the Fisher’s *F*-value was found to be 25.4683, suggesting that the model is significant for dye adsorption, presenting a 95% confidence level. Also, the the values of "Prob > *F*" that were found to be less than 0.05 show that the model terms have a strong/significant impact on the output response (Table 3). In this case, A, B, C, D, A2, B2, C2, and D2 are the significant model terms. Then, the high *p*-values found (0.9835, 0.8362, 0.9176, 0.2060, 0.5241, and 0.7410) for AB, AC, AD, BC, BD, and CD, respectively imply that the interaction terms are not such significant. On the other hand, the values found that were higher than 0.1000 indicate that the model terms are not significant. The factor coefficient confirms the influence of a fixed factor, while the two combined factors coefficients can reveal the interaction between them [49]. Table 3 indicates that independent variable, initial AB92 concentration (*F*-value: 161.7921) has the highest effect on the AB19 adsorption followed by initial pH (*F*-value: 116.8807), nanoadsorbent’s dosage (*F*-value: 19.2103) and contact time (*F*-value: 6.0019). The interaction between concentration and dosage gave a high *F* value (1.7476), which was higher than the other interactions. Also, it is found that the high value of the determination coefficient (*R*^2^ = 0.9597), which can reveal the fitness degree to the model, presented a high correlation degree between the predicted and actual/real output response. It is worth noting that when determination coefficient (*R*^2^) is close to 1, the model can be considered as significant presenting and satisfying all the terms of ANOVA [50]. The predicted *R*^2^ of 0.8277 is in reasonable accordance with the adjusted *R*^2^ of 0.9220. The signal to noise ratio is measured by the adequate precision and a ratio of 20.2425 indicates an adequate signal, because a ratio higher than 4 can be considered as appropriate. So, this model can be used to pilot the design space. The lack of fit (LOF) value was found to be 1.1039, which is significant.

The quadratic model was applied to elucidate the mathematical correlation between the independent and dependent variables. The mathematical model equation with the independent process parameters (pH, time, initial AB19 concentration, and Nd_2_O_3_ nanoparticles dosage) is presented in terms of the coded factors in relation to the AB92 percentage removal (response) and is given as Equation (5):

(5) $$\begin{array}{lll} Y & = & 88.7312-0.6913A-0.8133B+0.2803C-0.1566D+0.0014AB+0.0143AC+0.0071AD\\ & + & 0.0897BC+0.0442BD-0.0228CD+0.6961A^{2}+0.6373B^{2}-0.5413C^{2}-0.8165D^{2} \end{array}$$

where *A* = pH, *B* = concentration, *C* = Nd_2_O_3_ nanoparticles dosage and *D* = time.

**Figure 7:**
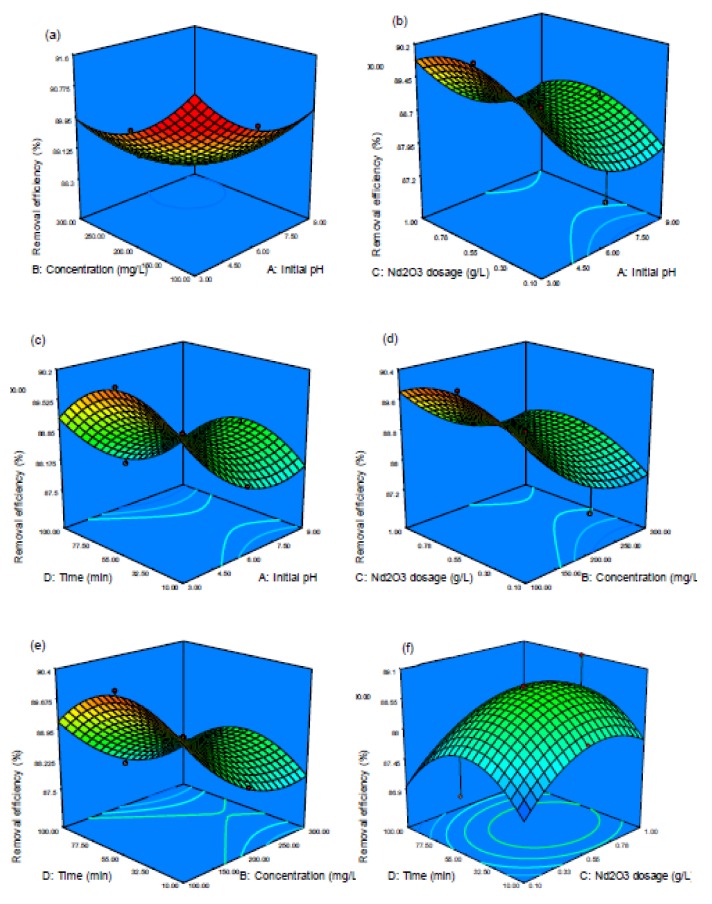
3D surface plot of the interactive effect of (**a**) AB92 concentration initial and pH, (**b**) Nd_2_O_3_ dosage and pH, (**c**) time and pH, (**d**) Nd_2_O_3_ nanoparticles dosage and concentration, (**e**) time and concentration, and (**f**) time and Nd_2_O_3_ dosage on AB92 adsorption percentage.

**Figure 8:**
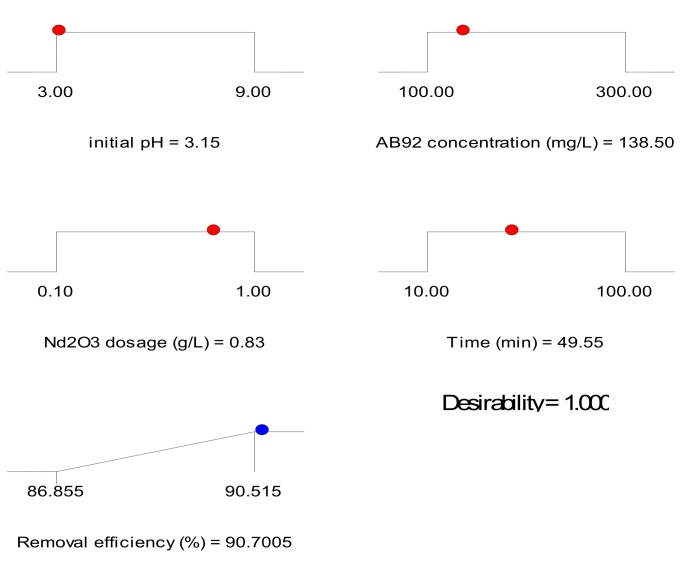
The desirability effect for AB92 adsorption on Nd_2_O_3_ nanoparticles.

**Figure 9:**
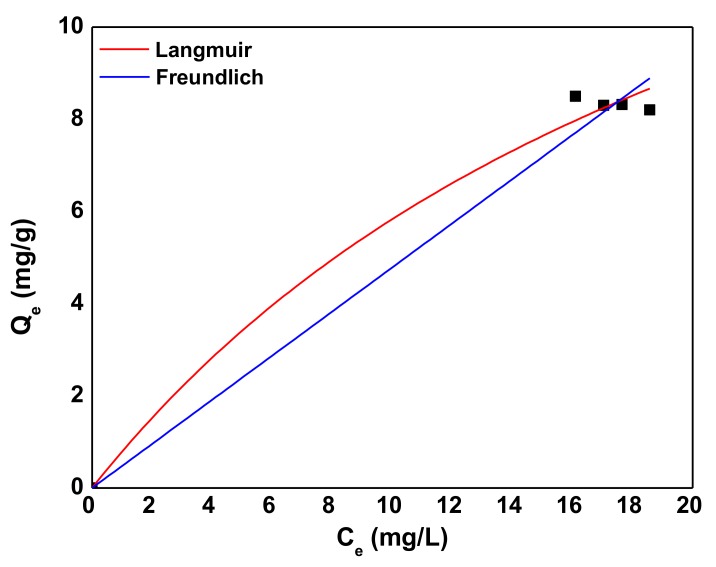
Adsorption capacity at various initial AB92 concentrations.

**Figure 10:**
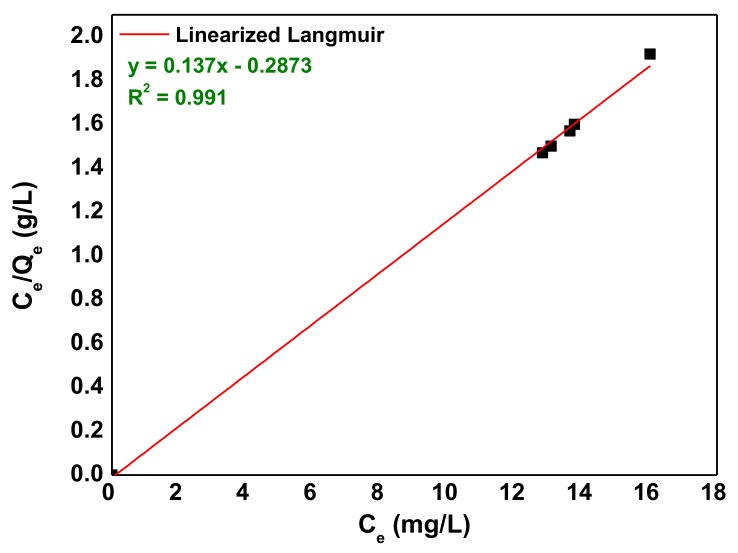
Linear Langmuir isotherm of AB92 adsorption onto Nd_2_O_3_ nanoparticles.

**Figure 11:**
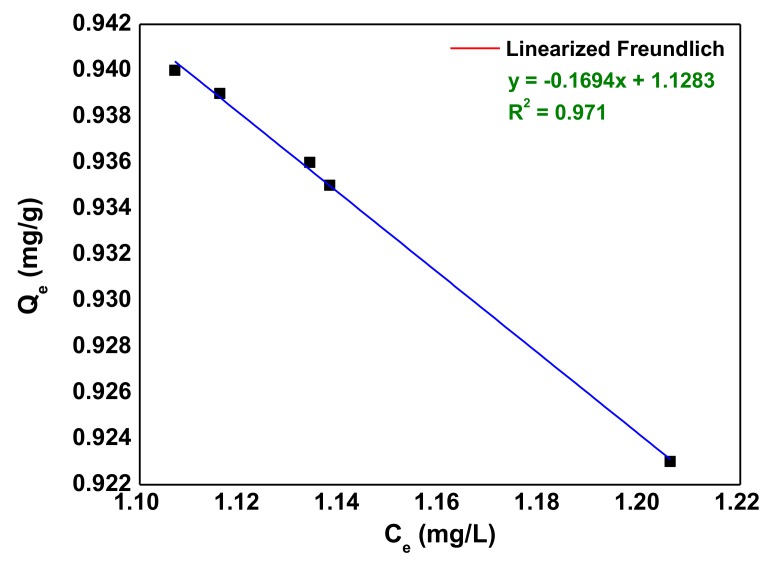
Linear Freundlich isotherm of AB92 adsorption onto Nd_2_O_3_ nanoparticles.

**Figure 12:**
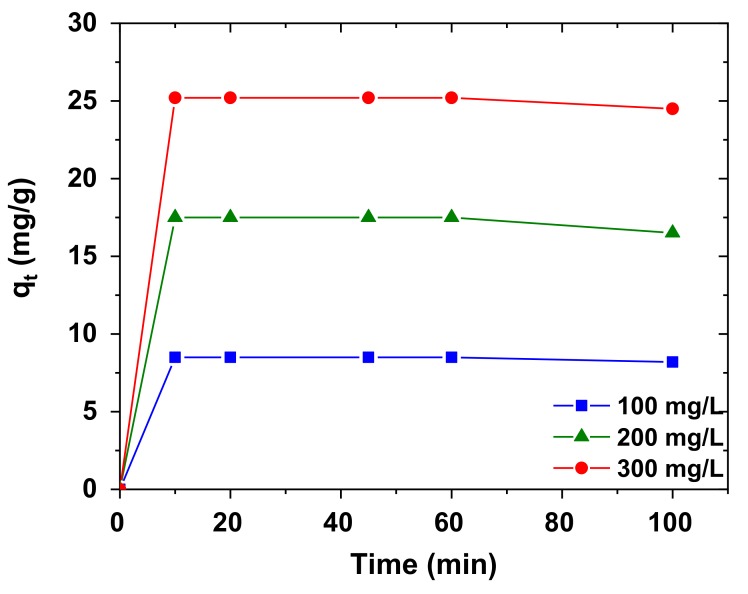
Adsorption kinetics of AB92 onto Nd_2_O_3_ nanoparticles.

The interactive effects of the operating parameters (independent variables) on the AB92 adsorption using Nd_2_O_3_ nanoparticles were studied by making three-dimensional (3D) response surface plots against any two independent variables while keeping the other parameters constant. The 3D surface plots of the output response (AB92 adsorption) from the interactions between the parameters are illustrated in Figure 7, Figure 8, Figure 9, Figure 10, Figure 11 and Figure 12. The pH, Nd_2_O_3_ nanoparticles dosage, AB92 concentration and time were studied in the range of 3–9, 100–300 mg/L, 0.1–1 g/L, and 10–100 min, respectively. It can be seen in Figure 7 that the other interactions apart from the interaction between time and dosage are not significant towards the removal of AB92. The pH is a very significant factor that affects an adsorption process [51,52,53]. The effect of pH on AB92 adsorption is linked to the solution pH and the functional groups exhibited by the Nd_2_O_3_ nanoparticles which will consecutively influence its surface charge [49]. The adsorption percentage was increased by decreasing the pH of the solution. However, it was observed that at higher pH (pH = 9), AB92 adsorption was reduced rapidly (Figure 7a–c). At pH higher than these value, the Nd_2_O_3_ nanoparticles had a negative charge (Figure 7). The adsorption of AB92 was more favorable in the acidic environment due to the presence of H^+^ on the adsorbent [54] and the electrostatic attractions between the negatively charged functional groups present on the anionic dye and the positively charged adsorbent (Nd_2_O_3_ nanoparticles) surface [55]. Figure 7a shows that optimal removal of 91.03% was achieved at a pH of 3.6 and concentration of 120 mg/L, 89.70% at pH 3.63 and time of 50.5 min (Figure 7c). Also, 89.75% optimal removal was reached at pH 3.6 and dosage of 0.69 g/L (Figure 7b). The reaction time is also vital in all research methods. Figure 7f shows the adsorption efficiency was increased by increasing the contact time and dosage of Nd_2_O_3_ nanoparticles. The efficiency was increased with increasing Nd_2_O_3_ nanoparticles dosage due to the availability of more active sites to trap the adsorbate (AB92); but beyond the optimal dosage and time, the adsorption efficiency was decreased. The removal of AB92 was improved rapidly with increasing contact time. The latter can be explained by the fact that high number of dye molecules does not have time to come in contact with nanoparticles to adsorb if the contact time is short [56]. The interaction between the initial AB92 concentration and pH has a negative effect on the adsorption process (Figure 7a). The adsorption efficiency declined with increasing initial concentration. Researchers have proved that at high concentrations, the removal efficiency is likely to decrease owing to the saturation of the adsorbent surface with the adsorbate contaminant [57]. The effect and significance of the interaction effects between the independent/process variables would have been lost if the experiments were done by using only the conventional means.

### 3.3. Optimization of Acid Blue 92 (AB92) Decolorization

Numerical optimization was executed through the Design expert software (Stat-Ease, 7.1 trial version) to define the optimal conditions for adsorption of AB92 on Nd_2_O_3_ nanoparticles. Adsorption efficiency of 90.70% was predicted at the optimal conditions of pH: 3.15, AB92 concentration: 138.5 mg/L, Nd_2_O_3_ nanoparticles dosage: 0.83 g/L and time: 49.55 min obtained. The validity of the predicted optimal values was proved by carrying out an experiment at these optimal conditions. Adsorption efficiency of 90.77% was obtained which is in agreement with the predicted adsorption percentage. Desirability value close to 1 shows the significance and acceptability of a model [58]. Figure 8 shows the desirability effect of the AB92 adsorption on Nd_2_O_3_ nanoparticles; the desirability of 1.000 confirms the applicability of the model and acceptability of the predicted output responses.

### 3.4. Adsorption Isotherms

In this research, the equilibrium data of AB92 adsorption on neodymium oxide nanoparticles were analyzed using the two most common isotherm models (Langmuir and Freundlich models). Figure 9 shows the typical equilibrium adsorption of AB92 onto prepared Nd_2_O_3_ nanoparticles at pH of 3, Nd_2_O_3_ nanoparticles dosage of 1 g/L, AB92 concentration of 100 mg/L and temperature of 289 K). The adsorption isotherm curve rises steeply at lower concentrations of AB92 and approaches to a plateau at higher concentrations.

The equilibrium data resulted were fitted to the non-linear Langmuir (Equation (6)) [59] and Freundlich (Equation (7)) [60] expressed by the following respective equations:

(6) $$Q_{e}=\frac{Q_{max}K_{L}C_{e}}{1+K_{L}C_{e}}$$

(7) $$Q_{e}=K_{F}C_{e}{}^{1/n}$$

where *Q*_e_ (mg/g) is the equilibrium dye concentration in the solid phase; *Q*_max_ (mg/g) is the maximum amount of adsorption; *K_L_* (L/mg) is the Langmuir adsorption equilibrium constant; *K_F_* (mg^1−1/n^ L^1/n^/g) is the Freundlich constant representing the adsorption capacity, *n* (-) is the constant depicting the adsorption intensity.

The parameters found after fitting were *Q*_max_ = 20 mg/g, *K_L_* = 0.04118 L/mg (*R*^2^ = 0.991) for Langmuir equation, while *K_F_* = 0.94095 mg^1−1/n^ L^1/n^/g, *n* = 0.08265 (*R*^2^ = 0.973) for the Freundlich equation.

However, many scientists also present isotherm models in linear expression. So, based on the ideal assumption of monolayer adsorption of adsorbate on the adsorbent surface, the Langmuir isotherm model is expressed in the linear form as follows [61]:

(8) $$\frac{C_{e}}{q_{e}}=\frac{1}{Q_{\max}}\cdot \frac{1}{K_{L}}+\frac{C_{e}}{Q_{\max}}$$

where the constants, qm, and *K*_L_ were determined by plotting (*C_e_*/*q_e_*) versus the equilibrium sorption, *C_e_* (Figure 10) and the calculated Langmuir parameters are recorded in Table 5.

The Freundlich adsorption isotherm model is expressed as [62]:

(9) $$\log\left(q_{e}\right)=\frac{1}{n}\log\left(C_{e}\right)+\log\left(K_{F}\right)$$

where *K*_F_ and 1/*n* are the Freundlich constants. The plot of log(*q_e_*) versus log(*C*_e_) (Figure 11) enables the determination of the isotherm, constants *K*_F_ and 1/*n* (Table 5). The isotherm parameters along with the regression coefficient, *R*^2^ are listed in Table 5.

The AB92 adsorption equilibrium data fitted into the studied isotherm models but the Langmuir was slightly better compared to the Freundlich isotherm with regards to its correlation coefficient, *R*^2^ (Table 5) with maximum adsorption capacity, *q_m_* of 7.299 mg/g under the experimental conditions. The favorability of an adsorption process is indicated by the magnitude of *n* [63]. The *n*-value of 5.903 determined for the Freundlich model lies within 1–10 (Freundlich threshold range) which suggests that the sorption process is favorable [63].

### 3.5. Kinetics of Adsorption

In order to further understand the adsorption process of AB92 onto Nd_2_O_3_ nanoparticles, the kinetics of the process was investigated. The experimental kinetic data were fitted into the Lagergren pseudo-first-order and Ho/Mckay pseudo-second-order kinetic models. The kinetics study was performed at pH of 3, Nd_2_O_3_ nanoparticles dosage of 1 g/L, and temperature of 298 K at different AB92 concentrations (100, 200 and 300 mg/L). The plot of *q_t_* versus *t* gives an excellent straight line relation for adsorption of AB92 on Nd_2_O_3_ nanoparticles (Figure 12).

**Figure 13:**
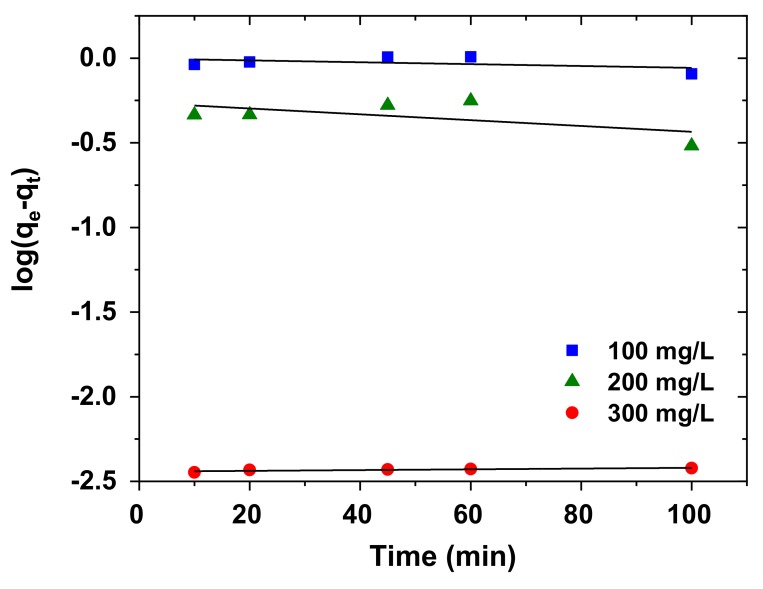
Pseudo-first order kinetic (Lagergren) model for AB92 adsorption onto Nd_2_O_3_ nanoparticles at 298 K.

The pseudo-first-order model is widely applied for the adsorption of liquid adsorbate on solid adsorbent on the basis of adsorption capacity at different time intervals (Figure 13). The pseudo-first-order rate equation is defined as Equation (10) [64]:

(10) $$\log\left(q_{e}-q_{t}\right)=\log\left(q_{e}\right)-\frac{k_{1}}{2.303}t$$

This equation is known as the integrated rate law for pseudo-second-order chemisorption reaction.

**Figure 14:**
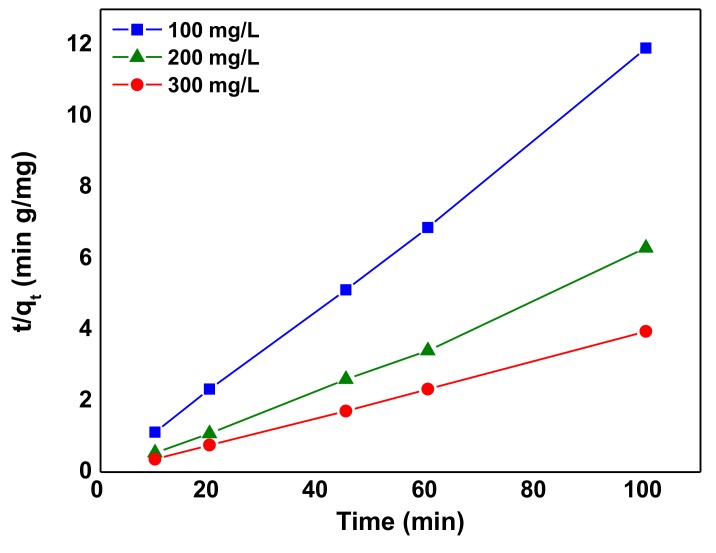
Pseudo-second order kinetic (Ho) model for AB92 adsorption onto Nd_2_O_3_ nanoparticles at 298 K.

The pseudo-second-order equation is given as Equation (11) [65,66]:

(11) $$\frac{t}{q_{t}}=\frac{1}{k_{2}q_{e}{}^{2}}+\frac{t}{q_{e}}$$

where *q_e_* and *q_t_* denote the amount of AB92 adsorbed per unit mass of the adsorbent at equilibrium and at time, *t* (mg/g). *k*_1_and *k*_2_ are the pseudo-first-order and pseudo-second-order rate constants (min^−1^), respectively. By plotting (*t*/*q_t_*) versus *t* (Figure 14), the constants *q_e_* and *k*_2_ were evaluated (Table 6). The R^2^ was used as the basis for choosing the appropriate kinetic model. The adsorption kinetic data was found to agree with the Ho kinetic model than the Lagergren (pseudo-first-order) model at all concentrations (Table 6) which suggests that the rate-limiting step is the chemisorption process [67]; this entails the sharing or exchange of electrons [68].

### 3.6. Thermodynamic Studies

The thermodynamic parameters including the standard Gibbs free energy (Δ*G*^0^), enthalpy change (Δ*H*^0^), and entropy change (Δ*S*^0^) for adsorption of AB92 onto Nd_2_O_3_ were calculated (Table 7) using the following equations [69]:

(12) $$\Delta G^{0}=-RT\ln K_{a}$$

(13) $$\Delta G^{0}=\Delta H^{0}-T\Delta S^{0}$$

where *R* is the universal gas constant (8.314 J/mol/K) and *T* is the absolute temperature in *K*. The thermodynamic parameter, Gibb’s free energy change (Δ*G*^0^), is calculated using *K_a_* obtained from the Langmuir isotherm.

**Figure 15:**
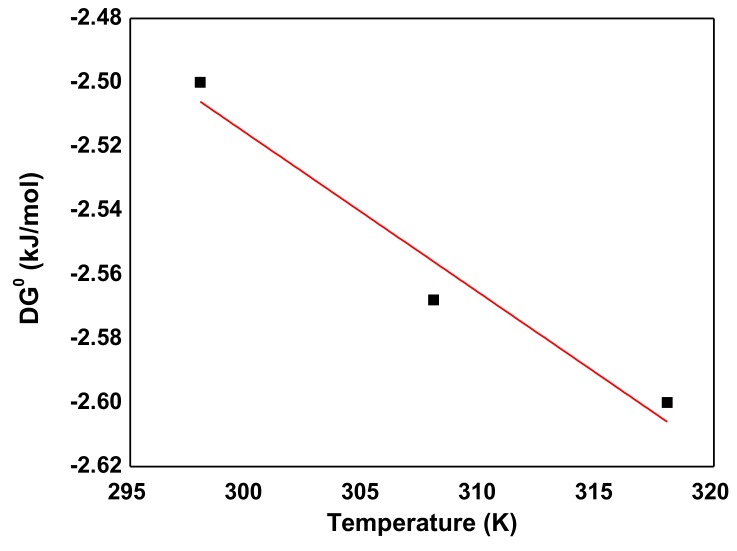
Free energy change versus temperature.

The negative Δ*G*^0^ values indicate that the adsorption of AB92 on Nd_2_O_3_ nanoparticle was spontaneous and favorable. The values of Δ*G*^0^ were found to decrease with increasing temperature (Table 7). For the calculation of Δ*H*^0^ and Δ*S*^0^ the plot of Figure 15 was used based on Equation (13). The positive value of Δ*H*^0^ indicates that the process is endothermic. According to Le Chatelier’s principle, increasing the temperature reduced the reaction rate and is followed by the reduction in the maximum adsorption capacity (*q_m_*). According to the results presented in Table 7, the Gibbs free energy values over –20 kJ/mol represent physical adsorption [58]. Δ*S*^0^ for the adsorption of AB92 by Nd_2_O_3_ nanoparticles is negative, suggesting that the degree of freedom at solid-solution level declines during the process of adsorption [70].

### 3.7. Reuse

**Figure 16:**
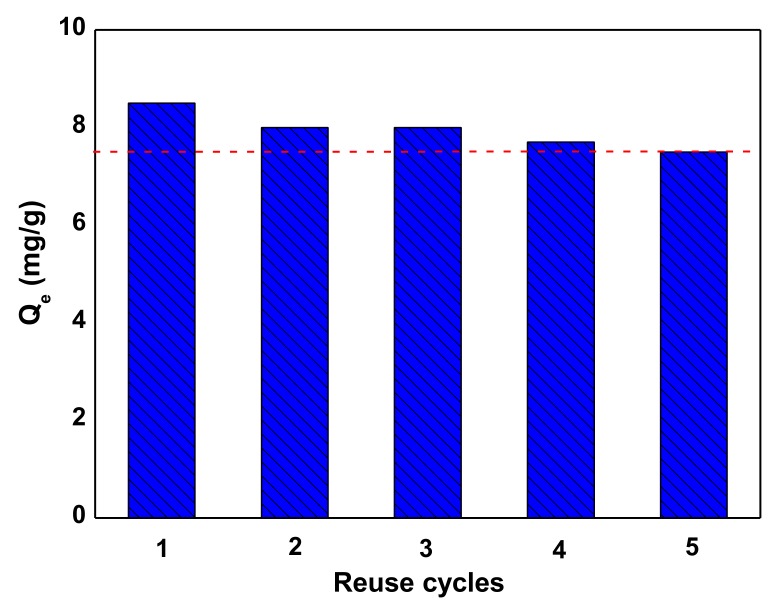
Cycles of reuse (adsorption-desorption) for the system Nd_2_O_3_/AB92.

Figure 16 illustrates the reuse potential of Nd_2_O_3_ after running the experiments in the same phase (aqueous) as that of adsorption experiments. The nanoparticles showed higher reusability, because it lost only 12% in adsorption capacity 5 cycles (8.5, 8.0, 8.0, 7.7, and 7.5 mg/g in the 1st, 2nd, 3rd, 4th and 5th cycle, respectively).

## 4. Brief Cost Analysis

### 4.1. Theoretical Approach

The most used adsorbent material is activated carbon; but as it will be discussed in the following it has drawbacks in reuse potential. The world demand for neat activated carbon is forecast to expand 5.2% per year through 2012 to 1.15 million metric tons. The consumption of activated carbons for industrial use has now become an indicator of development and environmental management efficiency. The per capita consumption of activated carbons per year is 0.5 kg in Japan, 0.4 kg in the U.S., 0.2 kg in Europe, and 0.03 kg in the rest of the world [71]. After the adsorbents are exhausted, they are either to be disposed off or regenerated for use. This depends upon the demand, the economics involved, and the kind of pollutant that was adsorbed. In many cases, spent adsorbents are to be treated as hazardous waste and need to be incinerated (which in many countries causes a set of environmental and societal problems) [72]. Exposure of spent adsorbents to ambient air may result in accumulation of heat due to adsorption of moisture and desorption of toxic adsorbates, creating hazardous conditions. Dumping spent adsorbents may also cause odour resulting in nuisance. The other option that industry can use is regeneration. Regeneration costs may equal to stabilization costs or just more than that, but if consumption of virgin adsorbent is reduced then multiple economic, industrial and environmental benefits can be gained. Extensive research has already been conducted regarding adsorption of pollutants onto various activated carbons, but investigations on regeneration remain scarce [73]. In many cases, the adsorbates may be a resource and need to be recovered or concentrated to earn recovery credits. Considering all the above arguments it is evident that spent adsorbent needs to be stabilized after being discarded. Because of high costs of production, stabilizing or proper disposal seem unlikely operations. Regeneration of adsorbents could prove double rewarding by stabilizing adsorbents and recovering valuable adsorbates, thereby minimizing demand for virgin adsorbents.

The main drawback of the already published adsorption studies is that their use is still in the laboratory stage mostly without pilot studies or commercialization. Limited attempts for detailed economic and market analyses are available [74]. Some attempts have been realized in the past at commercializing immobilized biomass dye biosorbents such as alga_SORB, AMT-bioclaim, B.V. Sorbex’s biosorbents and Bio-fix, but none have made a successful commercial entry in the market [75,76,77]. The main concept is not to extensively study the various fixed-bed adsorption papers in literature (and many of the parameters such as flow rate, bed volume, cross-sectional area, length, void fractions, adsorbent’s density, approach velocity, effective contact time, empty bed contact time, operation time, throughput volume, specific throughput, bed volumes), but to analyze and evaluate the first and fundamental principles of the use of green adsorbents.

In the case of the use of some green materials (mainly wastes) as a source for production of activated carbon, there is one serious problem: the regeneration cost. The costs of activated carbon adsorption are relatively high and the high costs limit its use in large-scale applications. The investment costs consist of the costs of equipment (instrumentation), pumps, pipes and monitoring systems). The operational costs depend mainly on the price of the adsorbent. The costs reduce when the adsorbent consumption per unit volume of treated wastewater reduces. In the adsorption process, electricity is mainly used for pumping the water and mixing the adsorbent suspension and for regeneration. In addition, the costs of regeneration and reactivation and the disposal costs of spent adsorbent must be taken into account when the total costs of adsorption are estimated. Spent adsorbent can include toxic substances and has to be treated as hazardous waste. The main conclusion of all above is that if an adsorbent is low cost but difficult to regenerate, it could not be economical and attractive for use [78,79].

As discussed above, the most important parameter determining the cost of the adsorption process to scale it up is the adsorbent and the regeneration cost. Furthermore, in a hypothetical scenario of using a green adsorbent produced from wastes, some other costs need to be taken into consideration. The cost of the adsorbent waste treatment consists of the cost of dewatering, transport and treatment by incineration or landfill and is estimated at 100 €/ton [79]. The adsorbent concentration needed to comply with the imposed discharge limits is influenced by the initial concentration of the pollutants in the water and the pollutant removal ability of the adsorbent. In systems containing only one pollutant and one adsorbent, an adsorption isotherm relates the adsorbents’ capacity to the pollutant concentration in the water at equilibrium conditions. Therefore, the appropriate adsorbent’s dosage has to be determined. For this reason, Kyzas et al. studied the use of spent coffee waste for dyes and heavy metal removal [80,81,82]. It was shown that 5 g/L was the best adsorbent dosage for the full decolorization of dyeing effluents. However, in each case, it is necessary to determine the environmental limits/regulations (discharge to aquatic systems) for each pollutant.

Another crucial factor regarding the operating cost of simulating adsorption procedure is the electricity, which is mainly used for pumping the water and mixing the adsorbent suspension. Vreysen and co-workers made a very useful cost estimation of the electricity required for an adsorption-flocculation system. The main equations used are the following: The suspension is mixed for 15 min at *G* = 821 s^−1^. The energy dissipation can be calculated from the formula:

(14) $$G={\left(\frac{P}{V\mu }\right)}^{1/2}$$

where *G* is the average velocity coefficient, *V* is the water volume, *μ* is the dynamic viscosity of water (8.9 × 10^−4^ Pa s) and P is the power required (Watt).

For the flow rate:

(15) V=QT

where *Q* is the flow rate (m^3^/h) and *T* is the time (h). Combining Equations (16) and (17) results in:

(16) $$P=G^{2}QT\mu [W]$$

or

(17) $$\frac{P}{Q}=\frac{G^{2}T\mu }{1000}[kWh/m^{3}]$$

**Figure 17:**
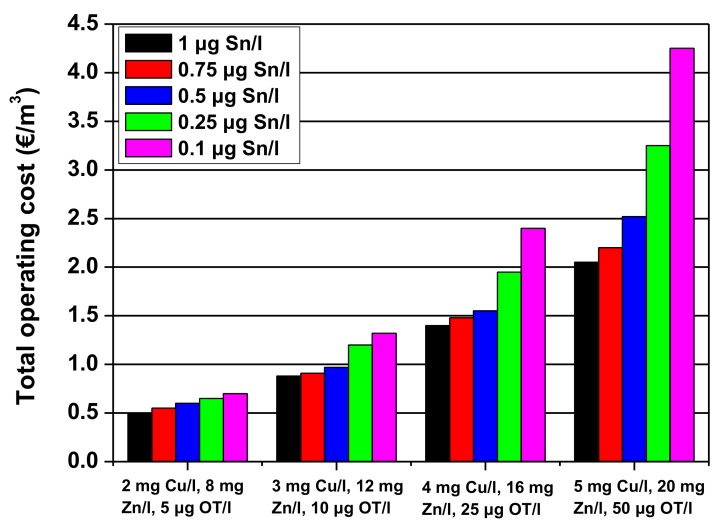
Total operating costs for 4 different wastewaters for 5 different organotin discharge limits. Reprinted with permission; Copyright Elsevier, 2008 [79].

The total operating cost consists of the sum of the adsorbent cost (including sludge treatment) and the electricity cost. Figure 17 shows a total operating cost estimation for 4 polluted wastewaters and 5 different organotin discharge limits as undertaken by Vreysen et al. [79]. The applied discharge limits for Cu and Zn were taken as 0.5 mg/L Cu and 2 mg/L. Zn in all cases.

From all above, it is clear that the most profitable use of green adsorbents is not those derived from activated carbon, but from agricultural waste. The use of those wastes untreated (just washed) seems to be even better. A possible scenario described below in order to compare is the following Table 8:

The parameters hypothetically are the same apart from the maximum theoretical adsorption capacity (*Q_m_*) and the estimated cost for the adsorbent production. In that case, 10, 5, and 3.3 kg of agricultural waste (AW), activated carbon (AC), and ACM are required for decontamination. However, in the case of AW the production cost is estimated to be zero. Instead, a factor of 0.5 is added. The production of AC is expected to be at least 4 times larger (electricity for pyrolysis etc.), while the respective for ACM is 6 times larger (pyrolysis, chemical modification etc.). It is clear that the order of profitability using the above adsorbents will be AW > AC > ACM. In another scenario, in which both other parameters vary, the superiority of green adsorbents will be even clearer.

In order to provide a more realistic scenario, for an average industry which treats and discharges 1 MGD (megagallons per day) as effluents (containing either dyes or metals), the approximate quantity of adsorbents can be calculated. In the case of textile industries, dye concentrations of 0.01–0.25 g/dm^3^ (= 10–250 g of dye per m^3^ of effluent) have been cited as being present in dye house effluents, depending on the dyes and processes used [83]. Therefore, 37.85–946.25 kg of dye (containing into the dyeing effluent) per day must be removed/adsorbed. Having as its basis the example of Table 9, 378.5–9462.5 kg of AW, 189–4731 kg of AC and 126–3154 kg of ACM are needed for the efficient treatment of effluent. However, as explained in the previous paragraph, the cost for the synthesis of AW in nearly zero. So, in any case this process can be characterized as sufficient. The same example for an average metal plating (chromium) with 2 MGD as effluent rate can be calculated mentioning that chromium concentrations of 0.5–270,000 g per m^3^ of effluent have been cited [84].

### 4.2. Application to the Present Nanomaterial

As was mentioned in the previous section, the wastewater treatment and the respective cost requirements are more or less similar for any textile wastewater treatment plants (WWTPs). However, there is big difference for the production of adsorbent material. To make a first attempt at the calculation of cost of the nanomaterial, it is mandatory to divide the cost in 3 major classes: (i) raw material cost (reagent, solvents, etc); (ii) energy cost for the synthesis; (iii) labor cost (personnel) [85]. Raw material cost prices for the study were obtained from publicly released catalogues. It should be noted that chitin and chitosan products can have very wide price ranges, depending on the quality of the final product. For example, chitosan prices might range between USD 10 to USD 1000 per kilogram. The costs calculated in this work are all translated to euros (€). The analysis took into account multiple market prices as provided by vendors all over the world. The raw material cost for each case included the adsorbent cost as well as the metal recovery cost. 

The energy cost corresponds to the energy spent for the various stages of the adsorption process. Electricity costs per KWh used are based on the average energy price in Greece for 2019 (0.194 €/KWh). This price was retrieved by the Hellenic Public Power Corporation S.A. [86]. Therefore, the energy cost in Euros has been estimated as the product of the amount of KWh spent and the price of 1 KWh in Greece.

Labor cost consists of the compensation of researchers participating in the project, with the addition of taxes and benefits. For the purposes of this study, the personnel required for the synthesis process comprises 1 researcher working for 1 work-day (i.e., 3 h). The average wages of the personnel were assessed based on information from Glassdoor [86], which maintains a rich database with employee wages per company and country depending on the position.

To calculate the recipe cost, Table 9 was drawn gathering all appropriate information. In this table, the method and duration used during synthesis is presented along with the relative instrumentation (reported energy consumption).

The equation used for calculations of energy cost is:

(18) $$E_{c}=P_{D}\cdot a\cdot t\cdot C_{c}$$

where *E_c_* is the energy cost (€), *P_D_* is the power consumed by device (kW), *a* is a load factor (if we use the device in full mode then *a* = 1, while for half mode *a* = 0.5, *t* is the usage of the device (*h*), *C_c_* is the energy cost (€/KWh).

After the appropriate calculations, the total energy cost for the procedure is 2.53 €. The raw materials cost was estimated form the catalogues of the suppliers. In particular, the cost was (i) 12.93 € for 0.06 mol of 2-hydroxy-1-netaldehyde; (ii) 1.56 € for methanol (60 mL); (iii) 2.19 € for Nd(NO_3_)_3_∙6H_2_O (with a molar ratio of 1:4); and 2.96 € for 1,4-diaminobutane (0.03 mol). So, in total for the raw costs was 19.64 €. But the latter cost was for 7.5 g of the prepared material. Therefore, the cost for the raw materials was 2.62 €/g. Similarly, the energy cost calculated in Equation (18) was 2.53 € for 7.5 g. But it is doubtful if it is correct to split this value for 7.5 g. So, it is decided to keep the whole energy cost as 2.53 €. Therefore, the total cost of production was 5.15 €/g.

## 5. Conclusions

The adsorptive removal of AB92 on neodymium oxide (Nd_2_O_3_) nanoparticles have been studied. The adsorption process was enhanced, modeled and optimized using CCD based on RSM. The influence of varying the pH (3–9), dosage (0.1–1 g/L), initial concentration of AB92 (100–300 mg/L) and contact time (10–100 min) on the adsorption process was examined. The adsorption process was found to fit best into the Langmuir isotherm and pseudo-second-order kinetic models. Also, the process was found to be spontaneous and favorable with increased temperature. Optimal conditions of pH 3.15, AB92 concentration of 138.5 mg/L, Nd_2_O_3_ nanoparticles dosage of 0.83 g/L, and contact time of 49.55 min were obtained which gave 90.70% AB92 removal. The values of the coefficient of determination, *R*^2^ (0.9596) and the adjusted *R*^2^ (0.9220) indicated that the process can be described by the RSM. The generated model was also found to be significant for AB92 adsorption at 95% confidence level. The prepared Nd_2_O_3_ nanoparticles have been applied successfully for the adsorptive removal of AB92 from its aqueous solution, modeled and optimized.

## Figures and Tables

**Table 1:** Experimental range and levels of the independent process parameters tested.

Factor	Independent Variables	Unit	Range and Level of Actual and Coded Values		
			−1	0	+1
*A* (*x*_1_)	Initial pH		3	6	9
*B* (*x*_2_)	Initial concentration	mg/L	100	200	300
*C* (*x*_3_)	Nd_2_O_3_ dosage	g/L	0.1	0.55	1
*D* (*x*_4_)	Time	min	10	55	100

**Table 2:** Experimental design matrix for AB92 adsorption on Nd_2_O_3_ nanoparticles.

Run	Initial pH (-)	*C*_0_ (mg/L)	Nd_2_O_3_ Dosage (g/L)	Time (min)	Experimental Adsorption (%)	Predicted Adsorption (%)
	*(A)*	*(B)*	*(C)*	*(D)*		
1	6	200	0.55	55	88.78	88.73
2	9	300	0.1	100	86.85	86.74
3	6	200	0.55	55	88.78	88.73
4	9	300	1	10	87.68	87.72
5	3	200	0.55	55	90.18	88.73
6	3	100	0.1	100	89.90	89.85
7	9	100	1	100	88.76	88.82
8	9	100	0.1	100	88.56	88.45
9	3	300	0.1	10	88.40	88.32
10	3	300	0.1	100	88.25	88.13
11	9	300	0.1	10	87.03	86.90
12	6	200	0.55	55	88.78	88.73
13	6	200	1	55	89.08	88.47
14	6	200	0.55	100	87.63	87.76
15	3	100	1	10	90.51	90.62
16	9	200	0.55	55	88.58	88.74
17	6	200	0.55	55	88.78	88.73
18	9	300	1	100	87.41	87.46
19	9	100	1	10	89.15	89.25
20	6	200	0.55	55	88.78	88.73
21	9	100	0.1	10	88.85	88.79
22	3	100	1	100	90.05	90.16
23	6	200	0.55	10	88.10	88.07
24	3	100	0.1	10	90.28	90.18
25	6	100	0.55	55	90.28	90.22
26	6	200	0.1	55	87.20	87.91
27	3	300	1	10	88.99	89.08
28	6	200	0.55	55	88.78	88.73
29	6	300	0.55	55	88.35	88.56
30	3	300	1	100	88.75	88.80

**Table 3:** Statistical models for AB92 adsorption.

Sequential Model Sum of Squares						
Source	Sum of Squares	df	Mean Square	*F* Value	*P* Value	Prob > *F*
Mean vs. Total	236,119	1	236119			
Linear vs. Mean	22.3634	4	5.5909	28.0665	< 0.0001	
2FI vs. Linear	0.1724	6	0.0287	0.1135	0.9937	
Quadratic vs. 2FI	3.7038	4	0.9259	12.5822	0.0001	Suggested
Cubic vs. Quadratic	1.0665	8	0.1333	24.9416	0.0002	Aliased
Residual	0.0374	7	0.0053			
Total	236,146.4	30	7871.546			
**Model Summary Statistics**						
Source	Std. Dev.	*R* ^2^	Adjusted *R*^2^	Predicted *R*^2^	PRESS	
Linear	0.4463	0.8179	0.7887	0.7743	6.1716	
2FI	0.5030	0.8242	0.7316	0.7612	6.5289	
Quadratic	0.2713	0.9596	0.9220	0.8277	4.7118	Suggested
Cubic	0.0731	0.9986	0.9943	0.8374	4.4467	Aliased

**Table 4:** Analysis of variance (ANOVA), lack of fit (LOF) test, regression coefficients and the significance of the response surface quadratic model of adsorption of the dye solution.

Source	Sum of Squares	df	Mean Square	*F* Value	*p*-value Prob > *F*	
Model	26.2396	14	1.8743	25.4683	<0.0001	significant
A-initial pH	8.6015	1	8.6015	116.8807	<0.0001	
B-AB92 concentration	11.9066	1	11.9066	161.7921	<0.0001	
C-Nd_2_O_3_ dosage	1.4137	1	1.4137	19.2103	0.0005	
D-Time	0.4417	1	0.4417	6.0018	0.0270	
AB	3.26E-05	1	3.26E-05	0.0004	0.9835	
AC	0.0033	1	0.0033	0.0443	0.8362	
AD	0.0008	1	0.0008	0.0111	0.9176	
BC	0.1286	1	0.1286	1.7476	0.2060	
BD	0.0313	1	0.0313	0.4254	0.5241	
CD	0.0083	1	0.0083	0.1133	0.7410	
A^2^	1.2554	1	1.2554	17.0588	0.0009	
B^2^	1.0524	1	1.0524	14.3002	0.0018	
C^2^	0.7592	1	0.7592	10.3157	0.0058	
D^2^	1.7272	1	1.7272	23.4695	0.0002	
Residual	1.1039	15	0.0736			
Lack of Fit	1.1039	10	0.1104			
Pure Error	0	5	0			
Cor. Total	27.3435	29				

*R*^2^: 0.9597, Adj. *R*^2^: 0.9220, Pred. *R*^2^: 0.8277, Adeq. Precision: 20.2425, C.V. %: 0.3058, PRESS: 4.7118, Std. Dev.: 0.2713, Mean: 88.7166.

**Table 5:** Isotherms parameters for the adsorption of AB92 onto Nd_2_O_3_ nanoparticles.

Langmuir		Freundlich		
*Q*_max_	*K_L_*	*K_F_*	*1/*n	*R^2^*
7.3	0.497	13.436	0.1694	0.9989

**Table 6:** Kinetics parameters for adsorption of AB92 onto Nd_2_O_3_ nanoparticles.

	Lagergren Isotherm			Ho Isotherm		
*C_0_*	*q_e_*	*k_1_*	*R^2^*	*q_e_*	*k_2_*	*R^2^*
100	0.95	0.0020	0.3547	15.847	0.027	0.9962
200	1.77	0.0048	0.5070	25.25	0.135	1.0000
300	262.30	1.1000	0.7862	8.39	0.129	0.9993

**Table 7:** Thermodynamic parameters for the adsorption system AB92-Nd_2_O_3_.

Temperature (K)	*C*_0_ (mg/L)	Δ*S*^0^ (J/mol K)	Δ*H*^0^ (J/mol)	lnK_a_	Δ*G*^0^ (kJ/mol)
298				−1.01119	−2.500
308	100 mg/L	−1.2	38.073	−1.00307	−2.568
318				−1.00307	−2.596

**Table 8:** Techno-economical comparison for green and non- green adsorbents.

Parameter	Green Adsorbents		Non Green Adsorbent
Adsorbent	Agricultural wastes (AW)	Activated carbon (AC)	Activated carbon (ACM)
Pollutants used	Dyes, Metals, Others	Dyes, Metals, Others	Dyes, Metals, Others
Modification	No	No	Yes
Adsorption capacity	100 mg/g	200 mg/g	300 mg/g
Mass of pollutant for removal	1 kg	1 kg	1 kg
Adsorption-desorption cycles	20	20	20
Loss of capacity after cycles	20%	20%	20%
Estimated cost for the adsorbent production*	0.5	2	3
Mass of adsorbent required	10 kg	5 kg	3.3 kg
Order of profitability	1	2	3

* This factor is used instead of using exact/unknown prices.

**Table 9:** Instrumentation and methods for nanomaterials synthesis.

Method	Duration (h)	Instrumentation
Soxhlet	12	Soxhlet Electrothermal (580 W)
Oven Drying	3	Oven Thermofisher (1450 W)
Stirring	3 (total)	Stirrer CAT M 6,1 (580 W)
